# Characterization of graphene oxide-*ziziphus* seeds and its application as a hazardous dye removal adsorbent

**DOI:** 10.1038/s41598-023-28924-2

**Published:** 2023-01-30

**Authors:** Badria M. Al-Shehri, Fatimah A. M. Al-Zahrani, Reda M. El-Shishtawy, Nasser S. Awwad, M. A. Sayed, Khalid Ali Khan

**Affiliations:** 1 Chemistry Department, Faculty of Science, King Khalid University, P.O. Box 9004, 61413 Abha, Saudi Arabia; 2 Research Center for Advanced Materials Science (RCAMS.), King Khalid University, P.O. Box 9004, 61413 Abha, Saudi Arabia; 3 Unit of Bee Research and Honey Production, Faculty of Science, King Khalid University, P.O. Box 9004, 61413 Abha, Saudi Arabia; 4 Chemistry Department, Faculty of Science, King Abdulaziz University, P.O. Box 80203, 21589 Jeddah, Saudi Arabia; 5 National Research Center, Dyeing, Printing and Textile Auxiliaries Department, Textile Research Division, Dokki, Cairo 12622 Egypt; 6 Physics Department, Faculty of Science, King Khalid University, P.O. Box 9004, 61413 Abha, Saudi Arabia; 7 Physics Department, Faculty of Science, Al-Azhar University, P.O. Box 71452, 71524 Assiut, Egypt; 8 Applied College, King Khalid University, P. O. Box 9004, 61413 Abha, Saudi Arabia

**Keywords:** Chemistry, Catalysis

## Abstract

The *zizphus* seeds are considered as a biomaterial residues that has been used for removing of organic industrial waste such as 2-((10-octyl-9,10-dihydroanthracene-2-yl) methylene) malononitrile (PTZS-CN) dye from aqueous solutions utilizing graphene oxide-*Ziziphus* (GO-*Ziziphus*). A batch study explored the impacts of various experimental circumstances, including solution pH, initial dye concentration, temperature, and contact time. General order, nonlinear pseudo-first order and pseudo-second order, elvoich model and intraparticiple diffusion were utilized to analyze the kinetic data. The adsorption kinetics of dye onto GO-*ziziphus* adsorption was best mentioned by nonlinear pseudo-first order. Similarly, the intra-particle diffusion plots revealed one exponential line throughout the adsorption process. The Freundlich, Dubinin-Radushkevich, and Langmuir models were employed to examine isothermal data. It provided the best fit of the dye adsorption isothermal data onto GO-*ziziphus* Freundlich models. Besides, the calculated free energies showed that the adsorption progression was physical adsorption. Thermodynamic calculations revealed that dye adsorption onto GO-*ziziphus* was exothermic and spontaneous. The combined results indicated that GO-*ziziphus* powder might be used to treat dye-rich wastewater effectively.

## Introduction

Global climate change and population growth have put pressure on water supplies. Wastewater management and potable water purification are crucial to sustain human society′s rapid development and mitigate environmental pollution and health hazards. One of the major problems concerning wastewater is the colored effluent^1^. The discharge from different range of industries such as textile industries^2^, paper and pulp industries^3^‏, dye industries^4^, pharmaceutical industries, tanner industries and paint industries are considered a wide variety of organic pollutants introduced into the natural water resources^5^. The discharge of colored wastewater is not only damaging to the aesthetic nature of the receiving streams but it may also be toxic to aquatic life. Contaminated water puts millions of people at risk of water-related illnesses^6^. According to UNESCO 2021 world water development report, diarrhea caused by unsafe drinking water, sanitation, and hand hygiene are the major causes for the death of about 829,000 people yearly^7^. Dye is one of the critical pollutants each year, almost 100,000 commercially accessible dyes and over 7105 tons of dyestuff are anticipated to be formed^8^. Removing dyes from waste has received much attention in the fight against water pollution.

Adsorption is one of the best processes for removing colors from aqueous solutions^9^. The most popular adsorbent for this purpose is activated carbon, which has a large surface area, a microporous structure, a high adsorption capacity, and a high level of surface reactivity. As a result, commercially available activated carbons are quite expensive^10^. This led to looking for a cheaper adsorbent for dye removal.

Agricultural wastes or lignocellulosic materials such as rice husk, sawdust, wheat straw, orange peels, baggase, peanut shells, etc. is an effective method due to their availability in abundance (naturally or as byproducts from waste industry), low cost, being environmentally friendly, feasibility for physical and chemical modification, and good adsorption capacity of heavy metal and dye^11^. *Ziziphus* seeds is one of the most important agricultural waste and excellent choice as adsorbent of heavy metal and dyes from wastewater^12–14^. It was found that *Ziziphus* seeds was the most effective adsorbent for many metals from aqueous solution such as cadmium ions (Cd^2+^)^15^ the removal reached 49.40 mg/g and 37.45 mg/g for the removal of Hg(II) ions from contaminated water^11^.

Graphene oxide is the oxidized form of graphene^16^, functionalized by a variety of active oxygenous groups in the planes and carbonyl and carboxyl groups at the edges^17^. GO is typically produced from flaky and synthetic graphites. The oxygenous functional groups on the GO surface (such as –OH, –COOH) are extremely beneficial to the hydrophilicity and high negative charge density, which are directly related to contaminant removal^18^.

The aim of the present work is to explore the possibility of converting *ziziphus* seeds which is one of commonly available waste materials widely spread in Saudi Arabia into graphene oxide. The produced material from *Ziziphus seeds* adsorbed the synthesized dye (PTZ-CN) with high maximum adsorption capacity of 29.5 mg/g. The dyestuff wastewater has been also treated using the produced materials.

## Materials and methods

### General

All experimental materials, such as solvents and chemical reagents with high purity, were purchased from Sigma-Aldrich Company. A Bruker Advance 600 MHz spectrometer was used to record 1H and ^13^C NMR spectra in CH_3_Cl-d_6_ solution. In addition, infrared spectra, mass spectrometry, and UV absorption spectra were performed by PerkinElmer, Agilent GC 7000 mass spectrometers, and Shimadzu UV–VIS Spectrophotometer, respectively.

### Synthesis and charatrization

#### Synthesis of under-studied organic dye

**Figure 1:**
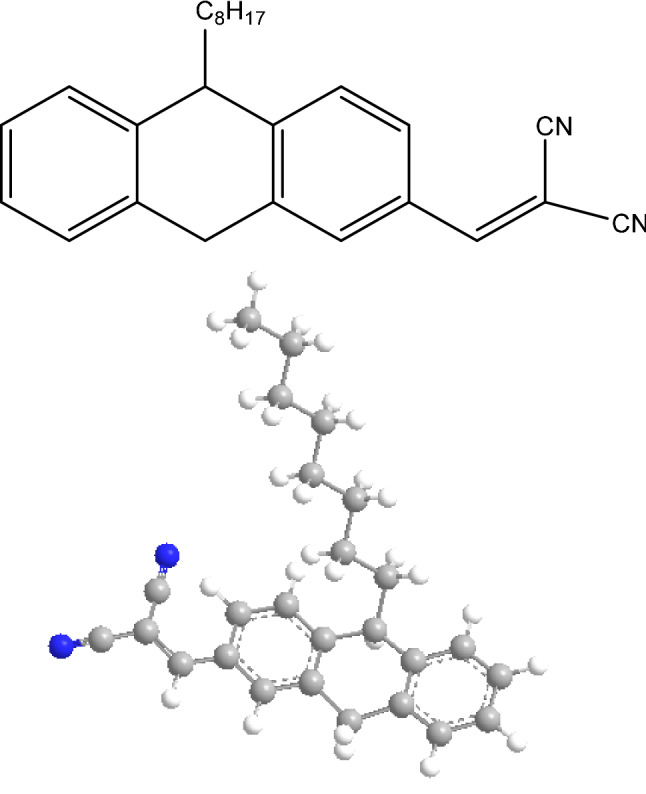
Chemical structure of synthesized PTZS-CN dye.

2-((10-octyl-9,10-dihydroanthracene-2-yl) methylene) malononitrile, abbreviated as (PTZS-CN). A mixture of 10-Octyl-10H-phenothiazine-3,7-dicarbaldehyde (1.18 g, 3 mmol), which was prepared, and malononitrile (0.395 g, 6 mmol) in basic ethanol solution (7 ml) was stirred at 25 °C for the whole night; it was purified and filtered by column chromatography to reach at 1.4 g with 74.6% yield. Figure 1 illustrates the chemical structure of the prepared organic dye. M.p. 58–59 °C; ^1^H NMR (600 MHz, CHCl_3_-d6) δ 3.908 (t, 3H, CH_2_-N), 6.892 (dd, 2H, J = 8.4 Hz, Ar–H), 7.013 (t, 2H, J = 7.2 Hz, Ar–H), 7.115 (dd, 2H, J = 7.8,1.2 Hz, Ar–H), 7.197(td, 2H, Ar–H), 7.508 (s,2H, C=C–H), 7.571(d, J = 1.8 Hz, 2H, Ar–H), 7.783 (dd,J = 7.8, 2.4 Hz, 2H, Ar–H). ^13^C NMR (125 MHz, CHCl_3_-d_6_) δ 14.29, 22.81, 26.91, 26.98, 29.38, 31.91, 48.43, 77.72, 113.77, 114.88, 115.12, 116.28, 123.20, 124.38, 125.20, 127.84, 129.75, 131.55, 142.69, 151.05, 157.55, IR, C-H aliphatic, 2925.00, 2851.31, CN 2225.48 cm^−1^, ESI–MS m/z [M]^+^calc 389.19 originate 387.1

#### Synthesis of adsorbent material

**Scheme 1:**
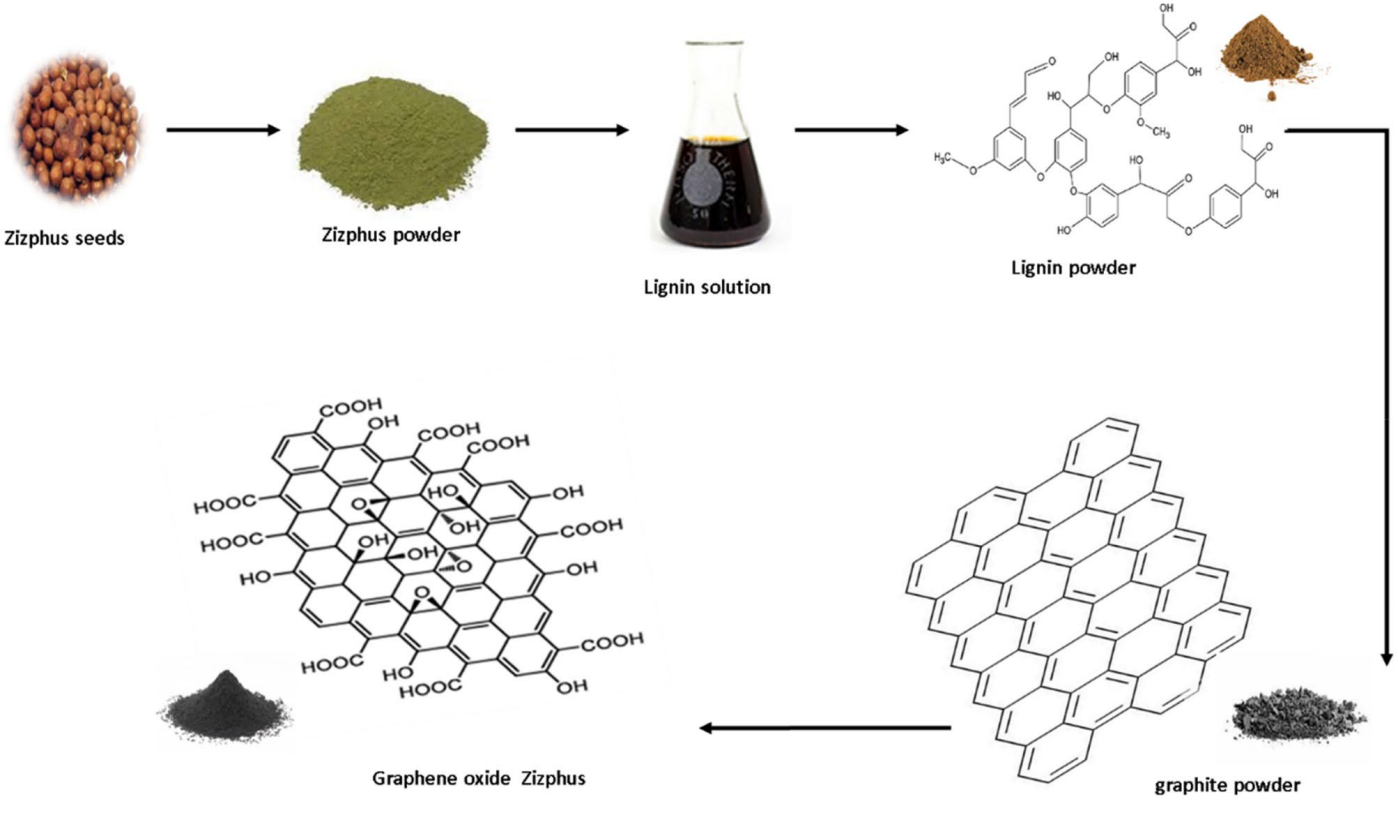
Proposed synthesis mechanism of graphene oxide ziziphus powder.

The three steps of preparation of graphene oxides-*ziziphus* were carried as follow as in the Scheme 1:

##### Conversion of *ziziphus* seeds to lignin

140 ml of 98% sulfuric acid was applied to 100 g of the *ziziphus* seed powder and stirred. The mixture was left to stand. The mixture was washed in a conical flask after 2 h and diluted with 3% sulfuric acid. Then, under reflux, the mixture was boiled for 4 h. The residue is washed and dried at 105 °C in an oven and then cooled and weighed as acid-insoluble lignin products^19^.

##### Conversion of lignin to graphite

In the present study, lignin was used as a carbon source obtained from *ziziphus* seeds, as in the first section, and iron (III) nitrate was used as a metal catalyst source in this research. A co-precipitation approach at room temperature was used to prepare iron with a 10% load in the iron-lignin precursors. Specifically, the lignin solution is prepared by applying 35 g of lignin with 50 ml of tetrahydrofuran in a beaker (250 ml) and mixing for 2 h. Meanwhile, the iron nitrate solution is prepared in a beaker (100 ml) by adding 41 g of iron (III) nitrate nonahydrate to 20 ml of distilled water and stirring the mixture to dissolve metal salt. After that, iron nitrate solution was mixed with lignin solution, and their mixture solution was stirred at 70 °C for two hours. The final mixture of iron and lignin was reserved for one day at 25 °C, then transported to the oven and dried at 150 °C for 24 h. Then iron-promoted lignin precursor was obtained and loaded into a quartz tubular reactor. The reactor was heated at 10 °C /minute to 600 °C with N_2_ gas, then passed CO_2_ gas at 10 °C /minute to 900 °C and reserved at 900 °C for 1 h. The oven automatically cooled down to room temperature by 10 °C/min. A few layers of graphite sample were collected from the quartz tubular reactor for Conversion to graphene oxide.

##### Conversion of graphite to graphene oxide –* ziziphus*

Hummer's modified method was used to oxidize graphite and eliminate catalysts from graphite to graphene oxide Specifically, 15 g of graphite was mixed with 200 ml 98% H_2_SO_4_ and 5.5 g NaNO_3_ into a 2000 ml flask and kept in the ice bath for cooling to 0 °C and stirred for 10 min. Then, 33 g of KMnO_4_ was mixed gradually, stirring continuously for 30 min. Then, the mixture was diluted with 1000 ml of distilled water and elevated to 75 °C for 20 min. The mixture was then preserved to stop the reaction with 500 ml of 10% H_2_O_2_. The overall response time from graphite to graphene oxide. was 60 min. Finally, 1.0 g of G.O. powder was gained from the G.O. solution after the freeze-drying process^20^.

### Determination of surface charge of adsorbent

The charge interactions of the adsorbent material with pollutant molecules are considered main factor that is control of the adsorption behavior. whereas, the point of zero charge (pH_PZC_) of graphene oxide *ziziphus* was detemined by dispersion 0.1 gm in the 50 ml of pH-adjusted aqueous solution with (0.1 M) of NaCl solution and (0.1 M) of HCl solution. The initial pH range of suspension was extended from 2 to 12. After 24 h, the final pH of solution was calculated.

The charge of adsorbent surface is neutral at value of pH_PZC_ while it was positive at lower than it. whereas, the surface was negative higher than pH_PZC._

### Adsorption study

All adsorption batches were run into glass Teflon-capped bottles shaken at 100 rpm by a water bath orbital shaker under constant pressure and temperature conditions. Typically, a fixed amount (gm) of adsorbent is placed in a fixed volume (ml) of adsorbate solution (PTZS-CN) at an initial concentration of C_0_ mg/l. The organic dye solution was made by dissolving a 1 g/L stock solution in deionized water.

The total concentration of dye in solution (C_o_) at (t) time (C_t_) by (mg/L) was assessed using a UV–Vis spectrophotometer double beam (Shimadzu, 1800, Japan). It ranged between 300 and 800 nm at applied lambda max (445 nm) (PTZS-CN) dye according to Beer-Lambert Low for test^21^.

The pH effect was investigated using a controlled concentration of NaOH and HCL reagents, a standard method for controlling the pH effect of adsorption tests in the literature. A Thermo Scientific pH-meter was used to assess the solution pH.

The preliminary results of the kinetic adsorption test showed that the required time to reach equilibrium conditions (contact time) was 40 min in all runs, and the equilibrium dye concentration (C_e_) was measured in (mg/L) per litre.

The adsorption percentage (%R) was determined by:

1 $$\% {\text{R}} = \left[ {\left( {{\text{C}}_{{\text{o}}} - {\text{C}}_{{\text{e}}} } \right)/{\text{C}}_{{\text{o}}} } \right] \times 100$$

The experimental data were expressed as a relationship between the equilibrium dye concentration (C_e_ mg/L) and the dye adsorption capacity q_t_ (mg/ g) at a given time and temperature. Furthermore, the following correlation was used to calculate the adsorption capacity:

2 $${\text{q}}_{{\text{t}}} = {\text{V}}\left( {{\text{C}}_{{\text{o}}} - {\text{C}}_{{\text{t}}} } \right)/{\text{m}}$$

Here,

(V) represents the volume of dye solution in (L).

(m) represents the weight of adsorbent in (g) (mg).

At contact time, dispersed (0.02, 0.05, 0.1 and 0.2 gm of adsorbent in 50 ml of dye solution at equilibrium pH) dye absorption and temperature were examined. A blank test of dye adsorption was also performed without adding an adsorbent to investigate the effect of the adsorbent.

## Results and discussion

### Charactrization of graphen oxide-ziziphus

**Figure 2:**
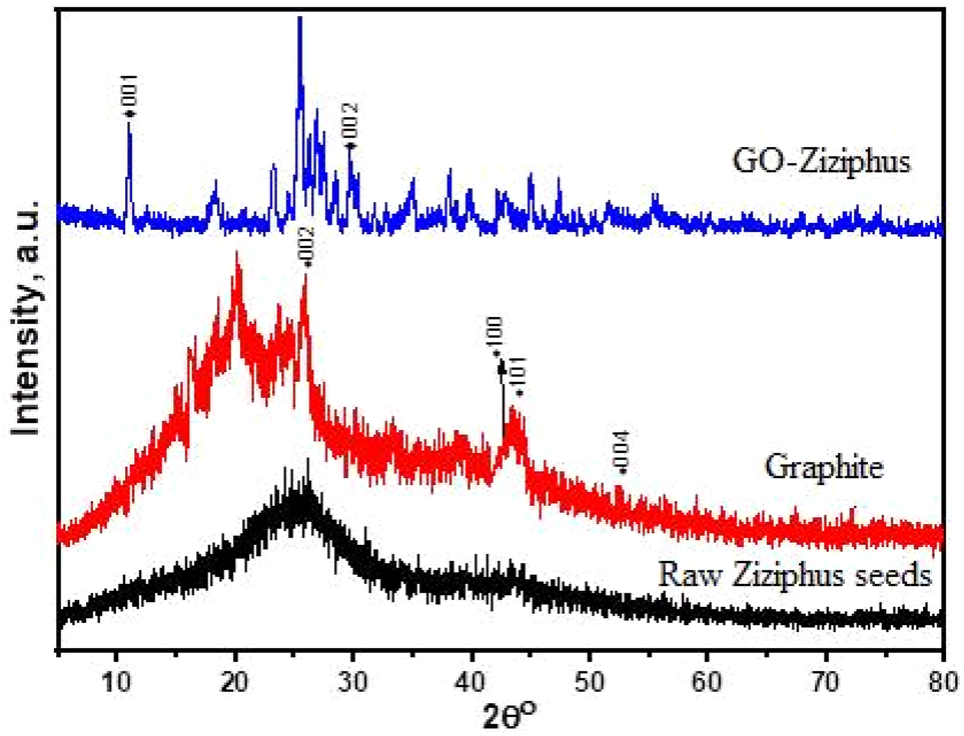
XRD patterns for raw *ziziphus* seeds, graphite and GO-*ziziphus*.

The crystalline structure of produced GO- *ziziphus* was investigated using XRD in the 2 = 10°–80° range, as shown in Fig. 2. The GO-*ziziphus* XRD data reveal a broad peak between 20° and 33° in angle (2). In addition, it lacks a basic horizontal line. These demonstrate that the majority of materials are amorphous^22^. The crystalline graphite structure may be seen in the peaks at (100) 42.53°, (101) 45.68°, and (004) 53.18°. The graphite XRD pattern shows a distinct peak (002) at 26.5° with an interplanar spacing of 0.334 nm. It suggests that graphite is a carbon substance with a high degree of orientation. The XRD pattern of GO-*Ziziphus* shifted from 26.5° to 11.66°, representing an interplanar detachment of 0.80 nm. The XRD pattern of GO-*ziziphus*, gives us a characteristic peak at 11.66°, representing for obtained GO phase, with still a characteristic peak for graphite, due to the washing step is still not enough to obtain clear graphene oxide. The introduction of distinct purposeful groups of oxygen throughout the oxidation process is due to the increase in interplanar distance of GO- *ziziphus*^23^. This increased distance between the layers could be linked to the presence of a number of oxygen-containing groups on the edge of each layer.

**Figure 3:**
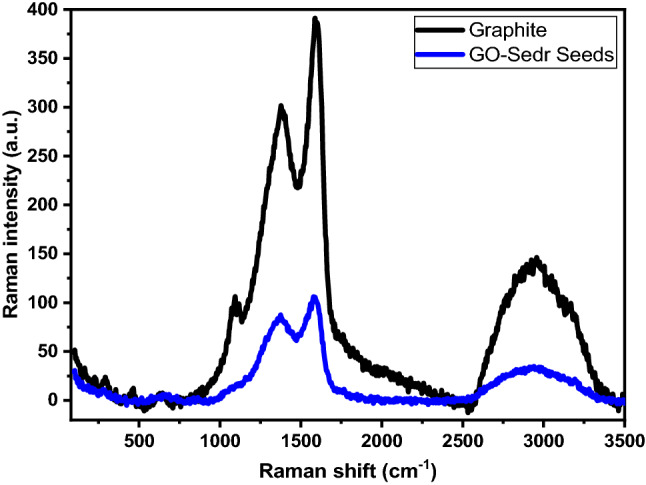
Raman-spectra for graphite and GO- *ziziphus* Seeds.

Their Raman spectra identified the prepared GO- *ziziphus* (Fig. 3), which allowed for the attention of the coupled and carbon–carbon double bonds that resulted in Raman peaks of high intensity. A G band represented the E_2g_ phonon of the sp^2^ C atoms at 1605 cm^−1^ and band D at 1353 cm^−1^, which resembles the breathing mode of k point phonons of A1g symmetry, describing the typical Raman spectra of GO- *ziziphus*.

**Figure 4:**
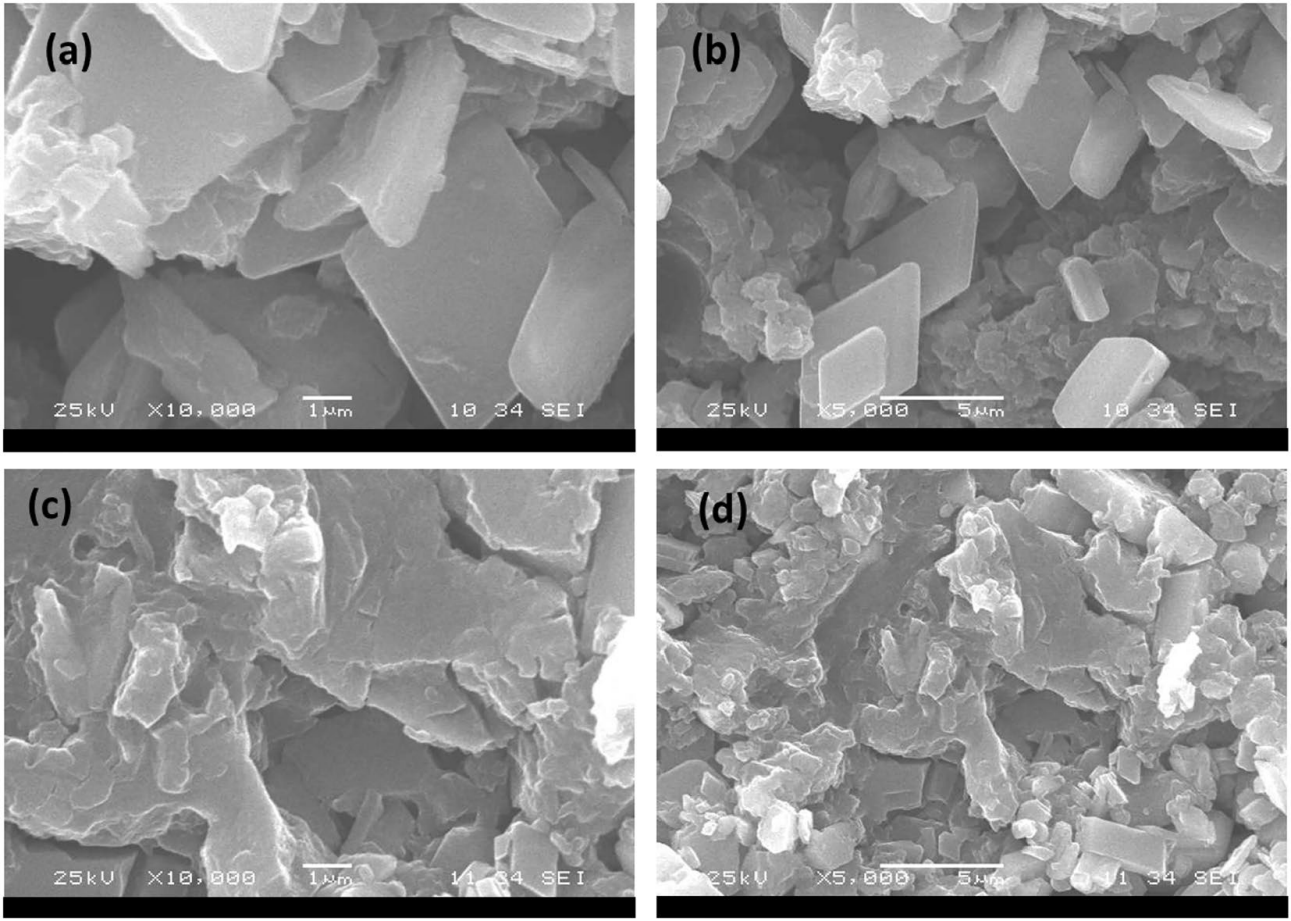
SEM micrographs for GO- *ziziphus* Seeds, (**a**,**b**) before, (**c**,**d**) after adsorption.

Subsection RDS, graphite, and graphene oxide SEM micrographs are displayed in Fig. 4. They had significant variances in terms of surface morphology. GO- *ziziphus* had a flat surface with no pores, according to SEM micrographs^24^. Compared to GO-*ziziphus*, however, the SEM pictures of GO-*ziziphus* revealed a high porosity level. Dyes can be up taken into pores produced on the surface of GO-*ziziphus*. The sheet-like structure of GO-*ziziphus* is visible in SEM micrographs. The boundaries of individual sheets could be detected from the SEM images, revealing wrinkled areas^25^. It is evident from the SEM micrographs that GO-*Ziziphus* has a multiple lamellar layer structure, and the edges of individual sheets can be eminent from the SEM micrographs.

**Figure 5:**
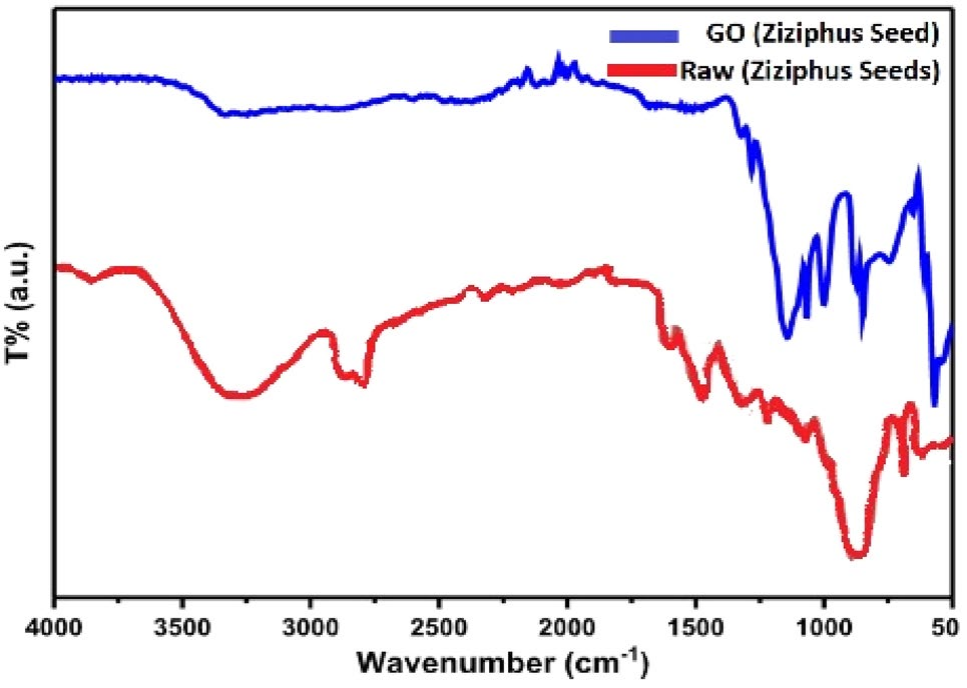
IR-spectra for GO-*ziziphus* Seeds.

The Fourier transform infrared spectrum which presented in Fig. 5, shows the distinguished functional groups of GO-*ziziphus* material. The first peak at 3320 cm^−1^ indicated to hydroxyl group (O–H), possibly due to water molecules. Besides, the peak at 1740 cm^−1^ indicated to stretching of carbonyl of (COOH) group. The third characteristic peak at 1620 cm^−1^ was denoted to the stretching carbon double bond of aromatic system. Furthermore, the C–O stretching of phenolic is responsible for the band at 1246 cm^−1^. The band describes the C–O stretching vibration of cellulose and hemicellulose appeared at 1028 cm^−1^. The rock vibrations (C–H) of cellulose were related to absorption at 870 cm^−1^.

## The adsorption performance of graphene oxide –Ziziphus (GO-Ziziphus) material

### Effect of various factors

**Figure 6:**
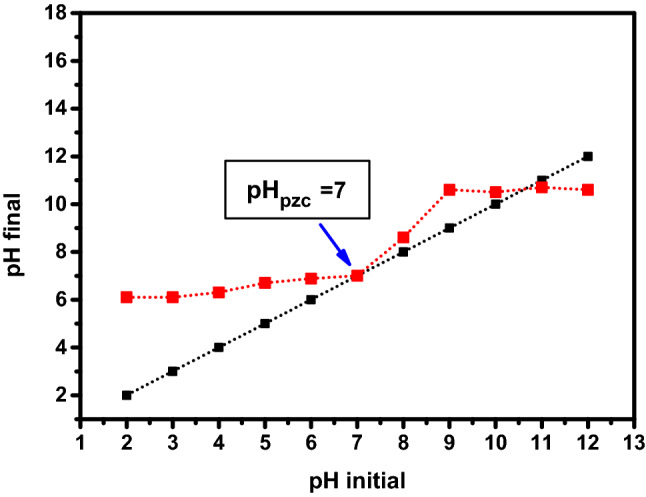
The point of zero charge (pH pzc) of graphene oxide-*ziziphus*.

The results of pH_PZC_ of surface of GO-*Ziziphus* is plotted in the Fig. 6 and the point of zero charge was at pH = 7 which refers to that at pH point the surface not charged while the charge of surface was positive at lower than pH = 7 and positive at higher than pH_PZC_.

This result meant the suitable pH value was at neutral pH which is agreement with a previous report^26^.

**Figure 7:**
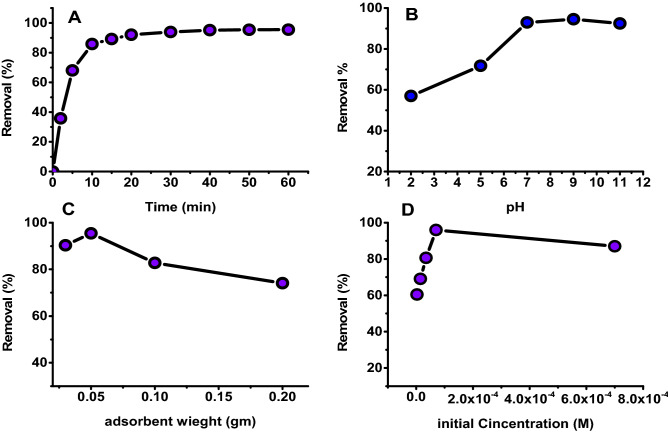
Effect of influential parameters on adsorption by GO-*Ziziphus* (**a**) contact time, (**b**) medium pH, (**c**) adsorbent weight, (**d**) initial concentration.

The time effect on the adsorption performance was investigated by added 0.05 gm of GO-*Ziziphus* into 50 ml of 7 × 10^–5^ M of dye suspension. The adsorption profile displayed in the Fig. 7a. The adsorption efficiency (R %) directly increase with the time increasing until reach to 95.5% at the equilibrium around 30 min. The rapidity of adsorption profile of organic dye onto GO- *Ziziphus* adsorbent indicates high adsorption performance of GO for these dyes in solution.

The adsorption efficiency was achieved in two ways: a relatively rapid step that lasted up to 30 min, followed by a slower one that lasted until the equilibrium state. The multi-types of active sites of the functional groups on the surface of graphene oxide are responsible of the high initial adsorption efficiency.

Solution pH is a critical factor in controlling adsorbents efficiency. Adsorption experiments were conducted at various pH levels by separating 0.05 gm of GO-*Ziziphus* into 50 mL of dye solution (C_0_ = 7 × 10^–5^ M) for 30 min at 298 K. Figure 7b shows the adsorption behavior with different pH values. The adsorption efficiency (R %) was initially increased with increasing of pH value up to pH 7 then the efficiency slightly decreased with increasing of basic medium. For a better understanding of the pH effect, at pH below pHpzc the adsorbent surface has a positive charge, and at pH above pH pzc, the surface has a negative charge. Therefore, this behavior of adsorption might be due to the multi-types of active sites of the functional groups on the surface of graphene oxide can bonded to organic dye via two ways. The first was the electrostatic attraction between the negative charge of the carboxylic groups on GO and the positive charge (–N + –Ar) of the dye molecule. The second was the π–π interaction taking place between the dye molecule and the aromatic rings of the GO^27^.

The adsorbent weight on the adsorption capacity was investigated using four different weights (0.02, 0.05, 0.1, and 0.2 gm) of GO-*Ziziphus* material. The fitted outcomes are mentioned Fig. 7c. The results show the order of dye removal efficiency of weights, with 0.05 gm having the best removal efficiency (95.4%) more than other weight.

The significance of initial dye concentration on the adsorption efficiency was analyzed in the range of 7 × 10^–4^, 7 × 10^–5^, 3.5 × 10^–5^, 1.5 × 10^–5^, and 7 × 10^−6^ M at pH = 7 and 298 K for 30 min with shaking. Figure 7d depicts the obtained results, showing that the adsorption efficiency increased with increasing dye concentration, the maximum removing of dye was obtained at 7 × 10^–5^ M. After that, the removing rate fall in with increase concentration which made it difficult for the GO-*Ziziphus* to adsorb the dye molecules. Maximum adsorption was obtained at 95.5%^28^.

### Adsorption kinetics

Five well-known kinetics non-linear models, such as pseudo-first order, and pseudo-second order, general-order, Elovich, and Intra-Particle-Diffusion models were utilized to detect the kinetics parameters. These kinetics models' nonlinear expressions can be used^29^.

3 $$q_{t} = q_{e} [1 - {\text{e}}^{{ - k_{1} t}} ] \quad {\text{non}}\;{\text{linear}}\;{\text{pseudo - first}}\;{\text{order}}$$

4 $$q_{t} = \frac{{k_{2} q_{e}^{2} t}}{{1 + K_{2} q_{e} t}} \quad {\text{Nonlinear}}\;{\text{pseudo - second}}\;{\text{order}}$$

5 $$q_{t} = q_{e} - \frac{{q_{e} }}{{\left[ {K_{n} \cdot \left( {q_{e} } \right)n - 1 \cdot t\left( {n - 1} \right) + 1} \right]^{{{\raise0.7ex\hbox{$1$} \!\mathord{\left/ {\vphantom {1 {1 - n}}}\right.\kern-0pt} \!\lower0.7ex\hbox{${1 - n}$}}}} }}\quad {\text{ non - linear}}\;{\text{General}}\;{\text{order}}$$

6 $$q_{t} = \frac{1}{\beta }ln\left( {\alpha \beta t + 1} \right)\quad {\text{ non - linear}}\;{\text{Elovich}}\;{\text{model}}$$

7 $$q_{t} = k_{diff} t^{1/2} + C\quad {\text{non - linear}}\;{\text{Intraparticle - Diffusion}}\;{\text{model}}$$

where (q_e_) indicated to the amounts of adsorbate dye at equilibrium, (q_t_) is indicated to the amounts of adsorbate dye at any time t in (mg/g). K_1_, K_2_ and K_n_ represent the pseudo-first-order (min^−1^), pseudo-second-order (g/mg. min) and general-order respectively.

**Figure 8:**
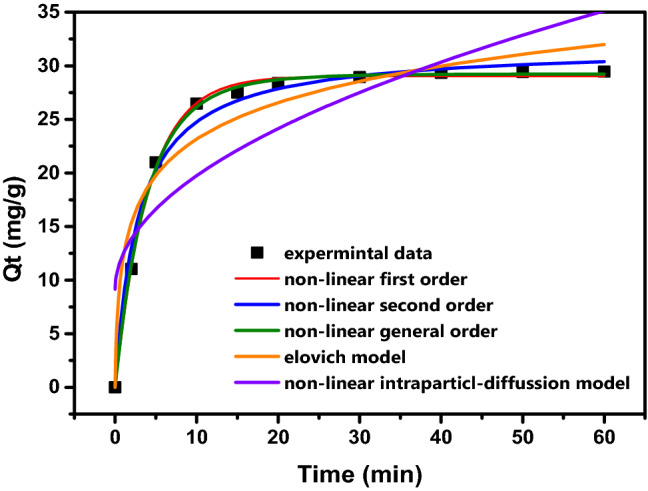
Adsorption kinetics models of nonlinear Pseudo-First-Order; nonlinear Pseudo-Second-Order, General order, Elovich and Intra-particle diffusion models.

The contact time was evaluated up to 60 min at an initial 7 × 10^–5^ M of dye. Kinetic curves (Fig. 8) shows that the adsorption process occurred extremely fast, with the equilibrium being reached at the 10 first minutes. The highly fast adsorption at the first minutes of the kinetic is due to the abundant available active sites that easily adsorb dye molecules on either surface and pore structures of the GO-*ziziphus*.

Table 1 shows the parameters of the kinetic models used to fit the experimental data. The models’ fitness was evaluated according to the adjusted determination coefficient (R^2^_adj_), and standard deviation of residues (SD). Lower SD and higher R^2^_adj_ values indicate a smaller difference between experimental and theoretical q values (given by the models) and therefore have the best suitable model. Based on these parameters, the nonlinear Pseudo-First-Order had the highest R^2^_adj_ and lowest SD values (see Table 1). The general order kinetics describes that the order of the adsorption process should be the same as that of a chemical reaction^30^. In a chemical reaction, the reaction order is measured experimentally^30^. The general order kinetic equation presents different values for n (order of adsorption rate) when the concentration of the adsorbate is changed, making it difficult to compare the kinetic parameters of the model^30^. So, t0.5 and t0.95 were used to compare the kinetics of adsorption of dye on GO-*ziziphus*^30^. t0.5 is the time to obtain half of saturation (q_e_) in the kinetic results, t0.95 was the time to obtain 95% of the saturation (q_e_). t0.5 and t0.95 were calculated and shown based on the best fitted model. Further evaluating the kinetic process, t0.5 was studied. The value was calculated from the best model (Nonlinear Pseudo-First-Order). These values correspond to the times (min) when 50% and 95% of saturation (q_e_) are attained, respectively^30^.Due to dye properties and chemical surface features, fast adsorption kinetics are observed from the values of t0.5 and t0.95. The adsorption procedure was further continued by establishing the contact times of 60 min (1 h). The established contact time was slightly higher than the t0.95 to ensure that the adsorption process had enough time to reach the equilibrium between GO-*ziziphus* and dye, because t0.95 was attained 95% saturation; the equilibrium was established in the condition of complete saturation of the adsorbent.

**Table 1:** Kinetics parameters of the adsorption of dye onto GO-*Ziziphus* adsorbent.

Kinetic models	
General–order model	
q_n_(mg g^−1^)	29.227
K_n_((g mg^−1^)^n−1^ min^−1^)	0.1729
n	1.1201
R^2^	0.99869
R^2^_adj_	0.99832
SD	0.21942
Pseudo first–order model	
q_1_(mg g^−1^)	29.03
k_1_(min^−1^)	0.245
R^2^	0.99827
$t_{0.5}$	2.83
$t_{0.95}$	8.97
R^2^_adj_	0.99805
SD(mg g^−1^)	0.18496
Pseudo second–order model	
q_2_(mg g^−1^)	31.827
k_2_(g mg^−1^ min^−1^)	0.0110
R^2^	0.98869
R^2^_adj_	0.98729
SD(mg g^−1^)	0.6605
Elovich model	
$\alpha$	53.03
$\beta$	0.202
R^2^	0.94434
R^2^_adj_	0.93738
SD(mg g^−1^)	39.63029
Intra-particle diffusion	
K_diff_	3.353
c	9.142
R^2^	0.72143
R^2^_adj_	0.93738
SD(mg g^−1^)	0.73666

Furthermore, the Intra-Particle-Diffusion model was utilized to elucidate the diffusion process and rate-determining step. It is established by Weber and Moris^31^ (Fig. 8).

The potted data between q_t_ and time appeared of presence of one straight line (Fig. 8). For describe of this result indicated to the rapid surface adsorption of pollutants with surface while intra-particle diffusion between pollutants molecules almost weak with a semi-stable rate. Likewise, when the results applied on the Elovich model the correlation coefficient was low (0.94) and the standard deviation was very (39.63) which was indicated to the nature of adsorption as physio-sorption mechanism on an energetically heterogeneous solid.

Figure 8 shows how the Weber-Moris Intra-Particle Diffusion model fits the experimental results. The figure is almost linear across the period investigated and does not cross through the source, indicating that adsorption is a one-step process^32–35^.

### Adsorption isotherm studies

Three different non-linear isotherm models were applied for the adsorption of dye on the GO-*ziziphus* adsorbent in order to predicting the adsorption capacity. Langmuir, Freundlich and Dubinin-Radshkevich models were applied at five temperature degrees, from 25 to 55◦C. The equations used for the Nonlinear isotherm models are:

8 $$q_{m} = \frac{{q_{m} kC_{e} }}{{1 + kC_{e} }}\quad {\text{Nonlinear}}\;{\text{Langmuir}}\;{\text{model}}$$

9 $$q_{e} = k_{f} C_{e}^{1/n} \quad {\text{Nonlinear}}\;{\text{Freundlich}}\;{\text{model}}$$

10 $$lnq_{e} = lnq_{max} - \beta \varepsilon^{2} \quad {\text{Dubinin - Radshkevich}}\;{\text{model}}$$

11 $$\varepsilon = RTln \left( {1 + \frac{1}{{C_{e} }}} \right) \quad {\text{Adsorption}}\;{\text{potential}}$$

12 $$E = \frac{1}{{\sqrt {2\beta^{1} } }}\quad {\text{Average}}\;{\text{energy}}$$

whereas K_.L._, K_.F._, $\beta$ and n are referred to the Langmuir isotherm constant, Freundlich constant, D-R constant and intensity factor, respectively. Farthemore, $q_{e}$ is the maximum amount of adsorbate at equilibrium (mg/g); $C_{e}$ is the concentration of adsorbate (mg/L);

(q _max_) referred to the theoretical adsorption capacity at saturation by mg/g, and ( R) is the gas constant (8.314 × 10^–3^ kJ/K mol). T is the temperature at which the reaction occurs (K). E is the average energy per adsorbate molecule transferred from the bulk (infinity) to the adsorbent's solid surface (kJ/mol).

**Figure 9:**
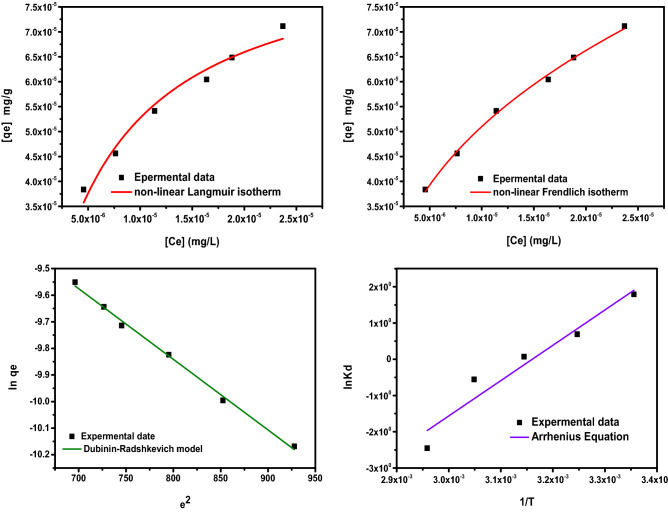
isotherm models of dye adsorption. (**a**) Langmuir (**b**) Freundlich (**c**) Dubinin-Radushkevich (D-R) (**d**) temperature effect.

The results which are displayed from isotherm models were listed in Table 2 and Fig. 9. According of correlation coefficient results of (R^2^) and standard deviation (SD) value of Langmuir and Freundlich models, the Freundlich isotherm model was the most suitable adsorption isotherm model for of dye removal from aqueous solution onto multi-layer adsorption of GO-*ziziphus*.

**Table 2:** Comparison of isotherm parameters of dye adsorption onto GO-*Ziziphus* .

Parameters	Isotherm models					
	Langmuir model		Freundlich model		D-R	
	q _max_ (mg/g)	34.72	Kf	0.0039	q _max_ (mg/g)	29.02
	K_L_ (L/mg)	149,465.8	n	2.6003	$\upbeta$	0.07933
	R^2^	0.9724	R^2^	0.9974	E(J/mol)	1.0175
	R^2^_adj_	0.96559	R^2^_adj_	0.9968	R^2^_adj_	0.99556
	SD	4.5723	SD	4.52016	SD	0.06303

Because it presented the highest R^2^ and lowest SD values. Freundlich’s model indicates that the adsorption process occurs on heterogeneous surfaces and active sites with different energies based on multilayer adsorption. For this model, an n value in this work was 2.60 that is between 1 and 10 and the adsorption was more favorable. The Freundlich model does not give maximum adsorption value (Q_max_), but the experimental adsorption capacity for the PTZ onto GO-*ziziphus* was 29.03 mg g^−1^. The physicochemical properties of GO-*ziziphus* can explain the efficiency of dye removal. Besides its very high surface area (2209 m^2^/g), it also presented a very high pore volume equal to 1.49 cm^3^/g. It is well known that pore volume plays a decisive role in the overall adsorption, as surface area does. Higher pore volume leads to higher sorption capacity. The GO-*ziziphus* molecule has a maximum diagonal length of 2.04 nm, a size that can be accommodated inside some pores of PTZ-CN.

Furthermore, the average energy of per dye molecule which transferred from solution to the surface of adsorbent was calculated Dubinin-Radshkevich model by plotting ln q_e_ vs ε^2^ in Fig. 9 and the results found 0.017 kJ/mol.

Comparison data of E value gave adsorption mechanism prediction nature, if which is greater compared to 8 kJ/mol, the adsorption is chemisorption; and if it is less compared to 8 kJ/mol, the adsorption is physisorption^36^. Here, the calculated value of free energy was E = 1.01 kJ/mol that referred to the adsorption mechanism of physical adsorption.

Additionally, the adsorption capacity (q) of PZC-CN onto GO-*ziziphus* was compared with other adsorbents reported in literature and is shown in Table 3. It can be seen from the table that GO-*ziziphus* shows the very high adsorption capacity with respect to other adsorbents, revealing that GO-*ziziphus* can be considered a viable adsorbent for the removal of dye from aqueous solutions.

**Table 3:** Comparison of monolayer adsorption capacity of different carbonaceous materials as adsorbents of pollutants with characteristics similar to those used in graphene oxide.

Adsorbents	Uses	Dosage (g·l^−1^)	pH	Contact time	T (°C)	Isotherm Model	Kinetic Model	Adsorption capacity mg/g	References
Activated carbon of *Ziziphus spina-christi* seeds	Removal Mn(II)	0.75	4	180 min	25	Freundlich isotherm	–	172.413	^37^
Activated carbon of *Ziziphus spina-christi* seeds	Removal Cu(II)	–	–	120 min	25	Langmuir isotherm	–	109.89	^38^
*Ziziphus* spina-christi L	Removal Hg(II) ions	8	6	60 min	30	Halsey model	Pseudo-second-order	37.45	^11^
*Ziziphus* jujube waste seeds	Removal (Cd^2+^)	1.8	7	40 min	–	Freundlich isotherm	Pseudo-second order	49.40	^15^
Graphene oxide- nanoplatelets	Safranin	0.50	–	120 min	30	Temkin isotherm	Pseudo second order	487.8	^39^
CSC-5GO	methylene blue	1000 mg L^−1^	12	180 min	25	Temkin	Pseudo-second-order	414.03	^40^
GO	Proflavine	25 mg/L	8.5	120	30	Freundlich	Pseudo-second-order	240	^41^
few-layered graphene oxide nanosheets	17b-estradiol	0.1 mg/mL	7		30	Langmuir isotherm	Pseudo-secondorder	149.4 mg/g	^42^
Graphene-coated biochar	MB	0.1 g	–	–	–	Langmuir	–	174	^43^
GNS/Fe3O4	MB	0.01 g	11	20 min	25	Langmuir	Pseudo-second-order	43.82	^44^
Fe3O4/GO hybrid	Bisphenol A	0.5 mg/L)	7	40 min	25	Freundlich	Pseudo-second-order	72.80	^45^
GO-*Ziziphus*	PTZ-CN	0.05	7	20 min	25	Freundlich	Pseudo-first-order	34	Present work

### Thermodynamic study

The thermodynamic section significantly calculated Gibb’s energy, enthalpy, and entropy change as sufficient parameters. The temperature influence was tested using 0.05 gm of GO-*Ziziphus* in 50 ml of dye aqueous solution at pH (7) and four diverse temperatures (25, 35, 45, and 55 °C).

These thermodynamic parameters can be expressed in the following way at first.

13 $$K_{d} = \frac{{q_{e} }}{{C_{e} }}\quad {\text{For}}\;{\text{equilibrium}}\;{\text{constant}}$$

14 $$\Delta G = - RTln K_{d} \quad {\text{For}}\;{\text{Gibbs}}\;{\text{energy}}\;{\text{change}}$$

15 $$\ln K_{d} = \frac{\Delta S}{R} - \frac{{\Delta {\text{H}}}}{RT}\quad {\text{For}}\;{\text{entropy}}\;{\text{and}}\;{\text{enthalpy}}\;{\text{change}}$$

“Where the (R) is the universal gas constant (8.314 × 10^–3^ KJ ^K−1^ mol^−1^); (T) is the absolute temperature (kelvin). The values of (∆H/R) and (∆S/R) were calculated from a slope and intercept as lnK_d_ vs 1/T, were plotted in Fig. 9.

According to the finding results in Fig. 9, increasing the temperature from 298 to 328 K reduced the adsorption capacity from 95.5 to 14%. That mean, the first indication of this behavior can be the adsorption is an exothermic process. Overall, increasing the temperature inhibited the adsorption capacity.

The calculated results are listed in Table 4. Results indicated that Gibb’s free energy became more positive with the increase in temperature and maximum was obtained at 328 K. This generalized the spontaneous nature of the dye adsorption onto GO-*Ziziphus* at room temperature. Additionally, enthalpy change was found to be − 84.33 J/mol suggesting the exothermic nature of the adsorption process. This is also in accordance with the isotherm results in which the better fit was obtained at lower temperatures. The entropy change showed a negative variation too.

**Table 4:** Thermodynamic parameters for adsorption of (PTZS-CN) dye (25 mg/L) at pH = 9 for 30 min.

t (°C)	T (K)	ln kd	∆G (J)	∆S (J)	∆H (J)
25	298	15.62	− 38.710	− 152.3097	− 84.33978
35	308	14.71	− 37.675		
45	318	13.69	− 36.203		
55	328	12.50	− 34.078		

### Reusability of GO-adsorbent

**Figure 10:**
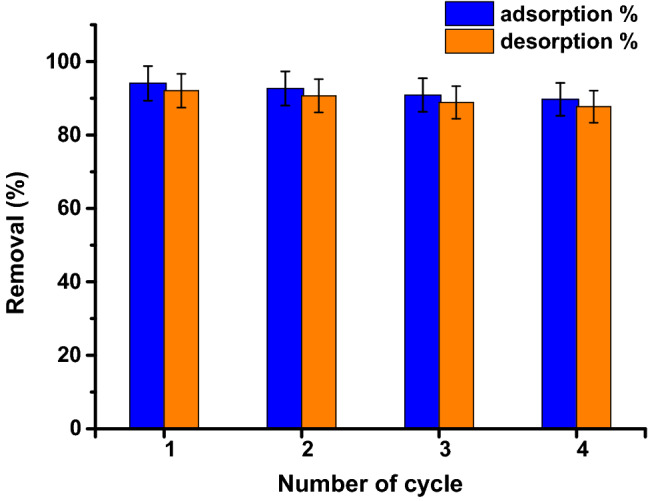
Desorption of dye and reusability of GO-*Ziziphus* for the adsorption.

The viability of any adsorbent on a commercial scale depends primarily on its recyclability. The reactivation of active sites of the adsorbent surface from adsorbed molecules is considered basic step to enter new adsorption cycle. In this study, the adsorbent GO-*Ziziphus* washed by acidic solvent followed by drying in incubator at 70 °C for 1 h. to desorbed dye molecules from adsorbent to the solution. The removal % results were depicted in Fig. 10 during continuous four cycles. The depicted diagram showed the fall in of adsorption efficiency from 95.5% in the first cycle to 88.3% at fourth cycle. The decreasing in the adsorption efficiency might be due to the partial coverage of GO-*Ziziphus* active sites by dye molecules which was not easy to desorbed from the adsorbent surface.

### The proposed mechanism of adsorption

**Figure 11:**
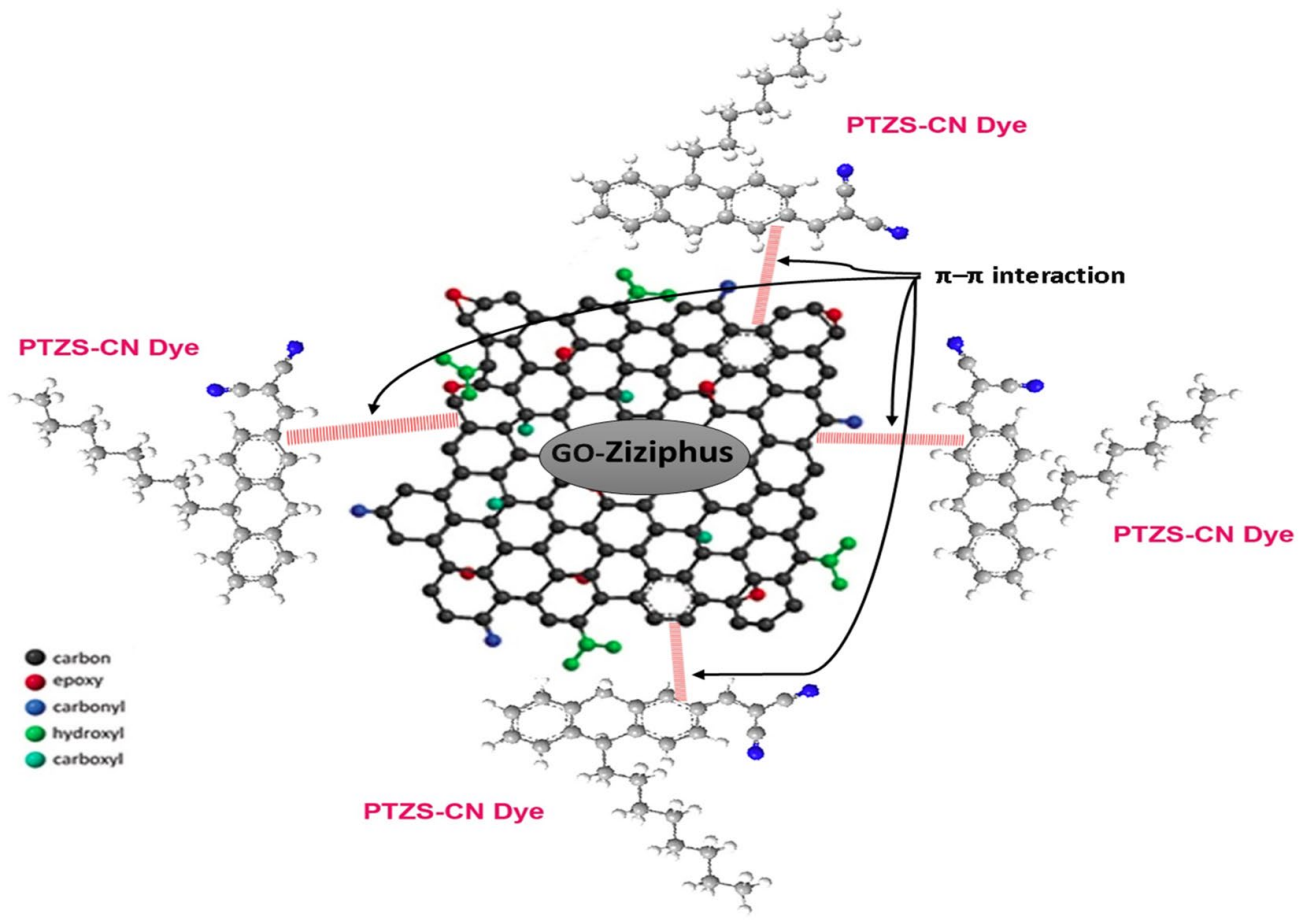
proposed mechanisms of PTZ-CN dye onto GO-*Ziziphus*.

There are various mechanisms might be explaining the adsorption behavior of Phenothiazine dye on the surface of graphene oxide. the main factors which controlled of the adsorption nature are electrostatics interactions (–N + –R), oxygen containing groups, π–π interaction, and photo degradation effect. At neutral pH, there is different types of binding can be occurring, one of them, ionic attraction between positive charged group from dye and (-OH) negative charged from GO-surface. Second, the interaction that might between dye and the epoxy (–O–), carboxyl (–OOC), and (OH) oxygen surface groups. Third, the π–π donor–acceptor interaction between double bonds of aromatic system of dye and GO-sheets. The last type might be play critical rule in this adsorption mechanism (Fig. 11).

## Conclusion

GO-*ziziphus* showed perfect adsorption capacity for organic dye removal. The maximum adsorption capacity (q_max_) of GO-*ziziphus* for organic dye calculated from the non-linear Freundlich isotherm was almost 30 mg/g at 298 K, which was the highest values of organic dye adsorption compared to the higher temperature. The kinetics and isotherm parameters can be well described by the non-linear pseudo-first order kinetic model and the freundlich isotherm, respectively. The adsorption process was exothermic and spontaneous. The high adsorption affinity of GO-*ziziphus* might be mainly due to the π–π donor–acceptor interaction between double bonds of aromatic system of dye and GO-sheets. The adsorption capacity was positive-affected by the increasing of solution pH. Moreover, the thermodynamic parameters of $\Delta H,\Delta G, and\;\Delta S$ are calculated in the process of dye removal. They are $\Delta H = 84.33 J$, $\Delta G < 0$, and $\Delta S$ < 0, respectively, which indicated that the adsorption process is exothermic, entropy decreased, and dominated by some complex mechanism, neither fully physical nor fully chemical adsorption. Also, adsorption kinetics was fitted with the non-linear pseudo-first order model, and the activation energy of this adsorption is 1.02 kJ/mol. As conclusion, GO-*Ziziphus* has several advantages, including low cost, availability of waste, and high efficiency. The study shows how waste material can effectively remove potentially dangerous waste.

## Supplementary Information

Supplementary Information.

## Data Availability

The authors confirm that the data supporting the findings of this study are available as a supplementary material.
