# Supplementary material for: Characterization of graphene oxide-ziziphus seeds and its application as a hazardous dye removal adsorbent
Source: Sci Rep. 2023 Jan 30;13:1631. doi: 10.1038/s41598-023-28924-2 (PMC9886902; doi:10.1038/s41598-023-28924-2)
Supplement: Supplementary file 1 — Supplementary Information. [file 41598_2023_28924_MOESM1_ESM.docx]

**Characterization of Graphene Oxide-ziziphus seeds and its application as a hazardous dye removal adsorbent**

**Badria M. Al-Shehri ^1,2,3*^, Fatimah A. M. Al-Zahrani ^1^, Reda M. El-Shishtawy ^4,5^, Nasser S. Awwad ^1^, Khalid Ali Khan^6,2,3.*^**

1. Chemistry Department, Faculty of Science, King Khalid University, P.O. Box 9004, Abha, 61413, Saudi Arabia
2. Research Center for Advanced Materials Science (RCAMS.), King Khalid University, P.O. Box 9004, Abha, 61413, Saudi Arabia
3. Unit of Bee Research and Honey Production, Faculty of Science, King Khalid University, P.O. Box 9004, Abha, 61413, Saudi Arabia
4. Chemistry Department, Faculty of Science, King Abdulaziz University, P.O. Box 80203, Jeddah 21589, Saudi Arabia
5. Dyeing, Printing and Textile Auxiliaries Department, Textile Research Division, National Research Centre, Dokki, Cairo 12622, Egypt
6. Applied College, King Khalid University, P. O. Box 9004, Abha 61413, Saudi Arabia.

***** Correspondence: [balshehre@kku.edu.sa](mailto:balshehre@kku.edu.sa) (BMA); [kkhan@kku.edu.sa](mailto:kkhan@kku.edu.sa) (KAK)

**
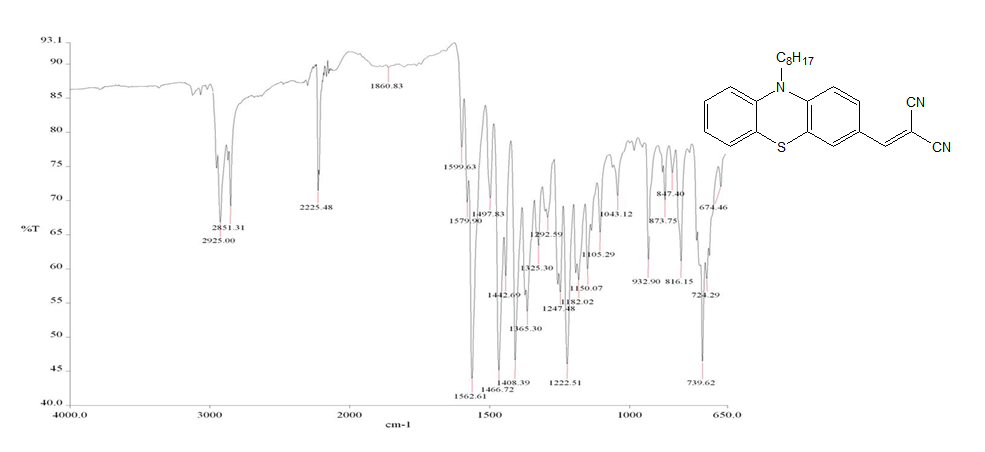
**

Figure S1. IR of ***2-((10-octyl-4a,10a-dihydro-10H-phenothiazin-3-yl)methylene)malononitrile***

**
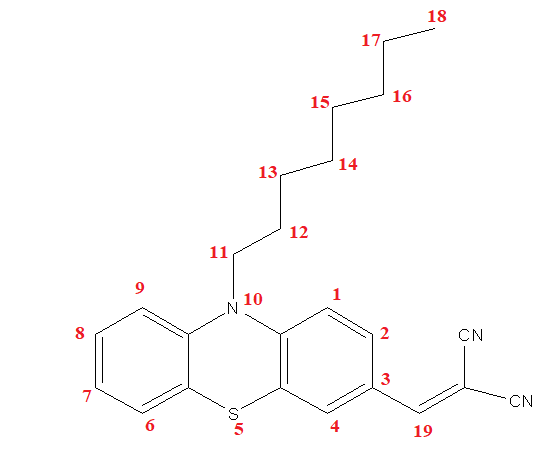

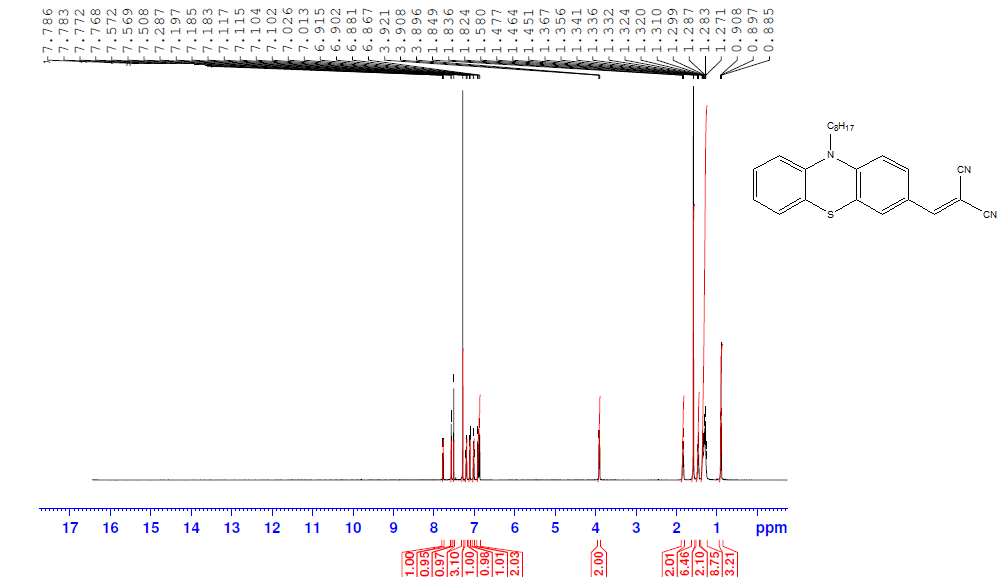
**

Figure S2. ^1^H NMR of ***2-((10-octyl-4a,10a-dihydro-10H-phenothiazin-3-yl)methylene)malononitrile***

**
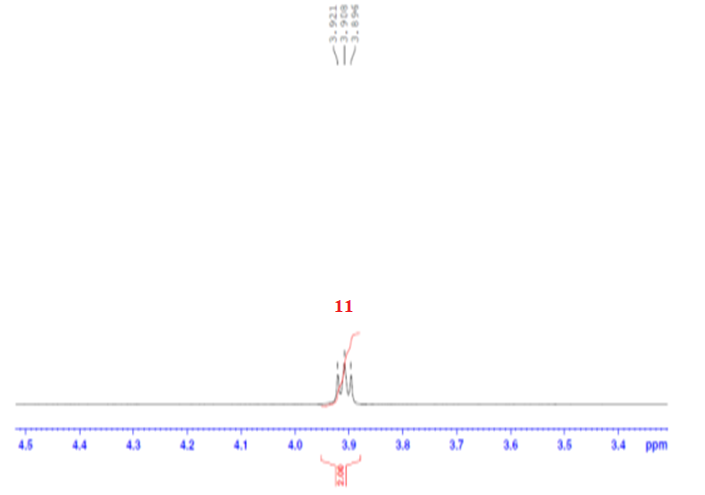
**

**
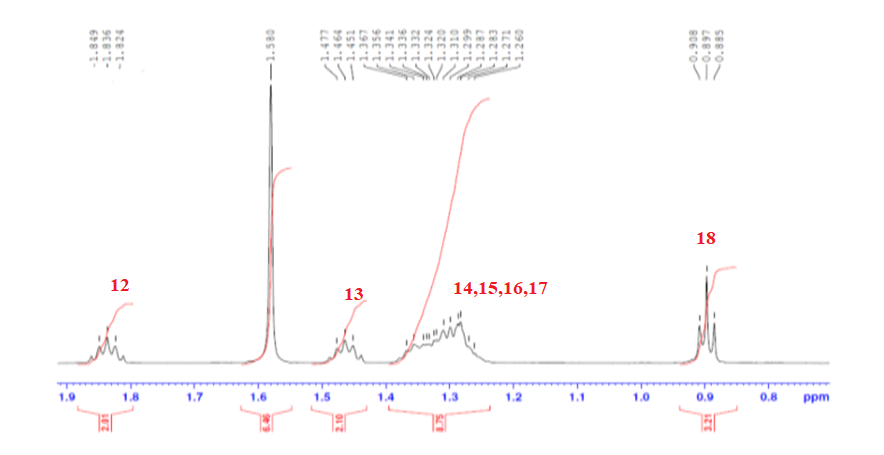
**

**
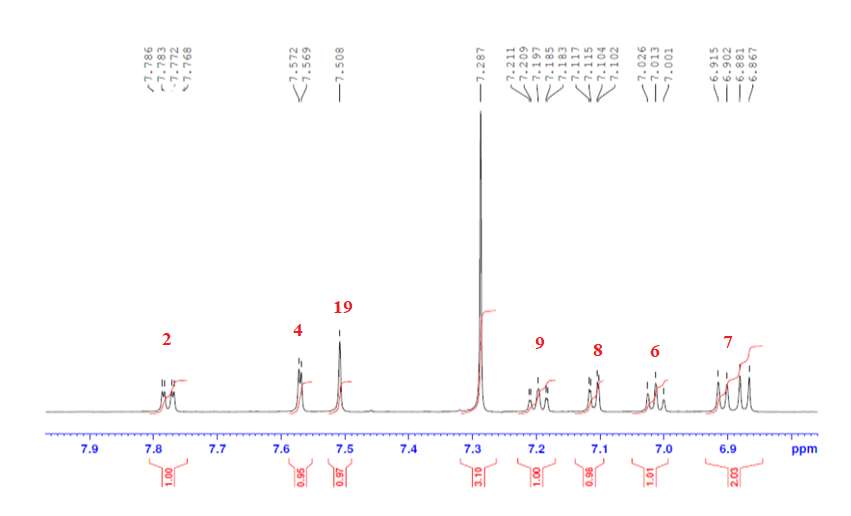
**

**
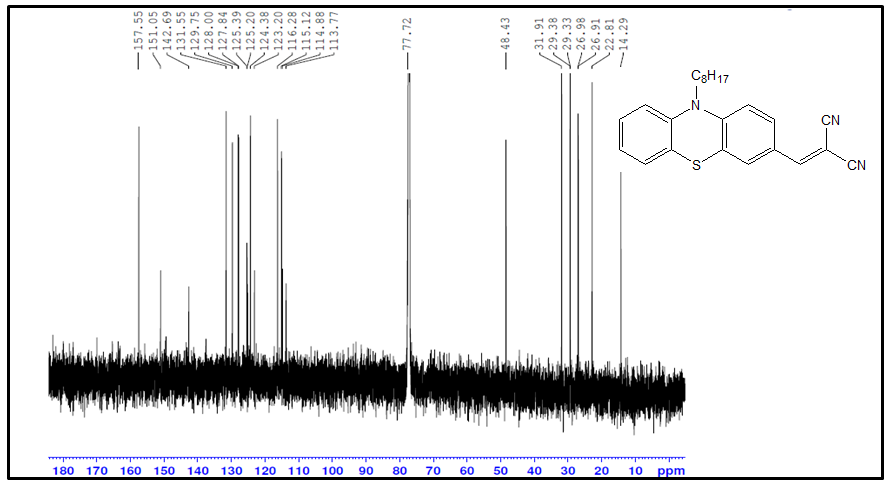
**

Figure S3. ^13^C NMR of ***2-((10-octyl-4a,10a-dihydro-10H-phenothiazin-3-yl)methylene)malononitrile***

***
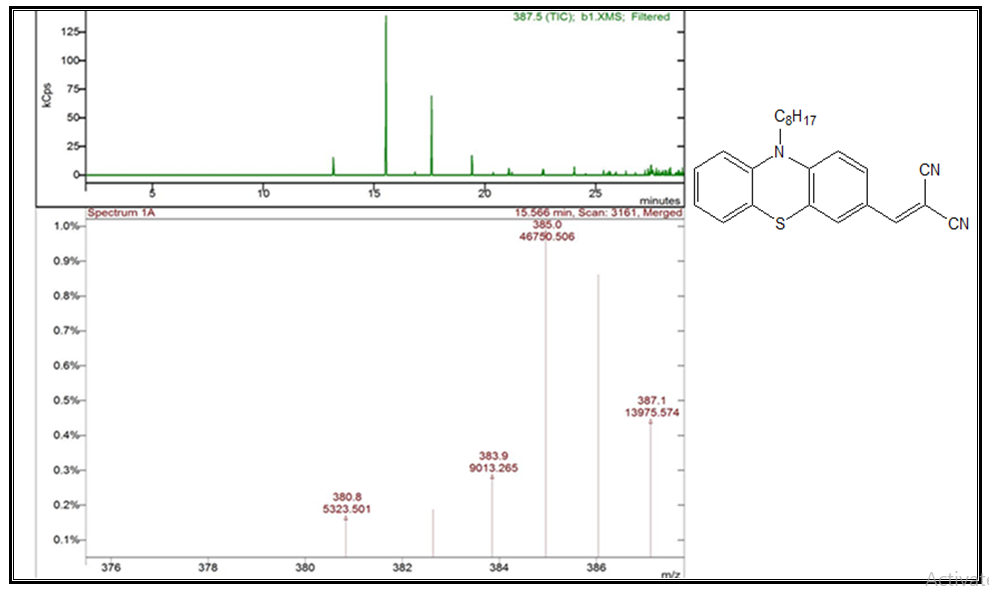
*** Figure S4. Mass of ***2-((10-octyl-4a,10a-dihydro-10H-phenothiazin-3-yl)methylene)malononitrile***
